# An analysis of policy success and failure in formal evaluations of Australia’s national mental health strategy (1992–2012)

**DOI:** 10.1186/s12913-017-2309-x

**Published:** 2017-05-30

**Authors:** Francesca C. Grace, Carla S. Meurk, Brian W. Head, Wayne D. Hall, Meredith G. Harris, Harvey A. Whiteford

**Affiliations:** 1 NSW Ministry of Health, 73 Miller St, North Sydney, NSW 2060 Australia; 2 School of Public Health, The University of Queensland, Herston, QLD 4006 Australia; 3 Policy and Epidemiology Group, Queensland Centre for Mental Health Research, Locked Bag 500, Archerfield, QLD 4108 Australia; 4 School of Political Science, The University of Queensland, Campbell Rd, St Lucia, QLD 4072 Australia; 5 Centre for Youth Substance Abuse Research, The University of Queensland, CYSAR K Floor Mental Health Centre, Royal Brisbane & Women’s Hospital Campus, Herston, QLD 4029 Australia

**Keywords:** Federal Government of Australia, Evidence-based reform, Mental health, Policy levers, Policy evaluation

## Abstract

**Background:**

Heightened fiscal constraints, increases in the chronic disease burden and in consumer expectations are among several factors contributing to the global interest in evidence-informed health policy. The present article builds on previous work that explored how the Australian Federal Government applied five instruments of policy, or policy levers, to implement a series of reforms under the Australian National Mental Health Strategy (NMHS). The present article draws on theoretical insights from political science to analyse the relative successes and failures of these levers, as portrayed in formal government evaluations of the NMHS.

**Methods:**

Documentary analysis of six evaluation documents corresponding to three National Mental Health Plans was undertaken. Both the content and approach of these government-funded, independently conducted evaluations were appraised.

**Results:**

An overall improvement was apparent in the development and application of policy levers over time. However, this finding should be interpreted with caution due to variations in evaluation approach according to Plan and policy lever. Tabulated summaries of the success and failure of each policy initiative, ordered by lever type, are provided to establish a resource that could be consulted for future policy-making.

**Conclusions:**

This analysis highlights the complexities of health service reform and underscores the limitations of narrowly focused empirical approaches. A theoretical framework is provided that could inform the evaluation and targeted selection of appropriate policy levers in mental health.

## Background

### Evaluation: methodological challenges and opportunities

The development of an evidence base for mental health reform requires an understanding of which instruments of policy, or policy levers, are likely to succeed or fail, and why. Evaluations of past initiatives can inform future policy development, however the undeniable importance of this task is challenged by the complexities of policy analysis [1, 2].

Within health services research, evaluation often focuses on outcome or impact measures, as a means of assessing the balance sheet (i.e. the goods and services produced by an intervention compared with the initial resource inputs) [3]. Selected indicators (usually quantitative), are measured before and after the intervention, and are (ideally) compared with control conditions, to identify the impact of the intervention in terms of its original objectives and any longer-term changes in the target group [3]. In theory at least, under this paradigm, ‘success’ and ‘failure’ can be assessed in terms of (statistically significant) favourable or unfavourable outcomes. These types of evaluations can be applied to specific programs or initiatives or to broader policies and reforms, depending on the existence of appropriately sourced data.

There is a history of advocacy and use of these types of evaluation in policy piloting and policy experiments, as one approach to evidence-based policy-making. However, many scholars have pointed to their limitations [4, 5]. Insights from policy studies suggest that the process of identifying policy successes and failures is complex, multidimensional, subjective and inescapably political [5, 6]. One element of complexity arises from the different roles, responsibilities and relationships between levels of government. These present both a practical challenge to policy implementation and empirical challenges for evaluation.

Australia operates under a federated system of government. One key feature is a vertical fiscal imbalance arising from Federal Government control over tax revenues, allowing increased Federal Government influence over health policy and service delivery functions traditionally reserved for the States and Territories [7]. In the case of mental health, for example, the use of Federal grants and other monetary incentives is tied to compliance with agreed standards (e.g. National Minimum Data Set and National Standards for Mental Health Services) [7]. There is, however, a prevalence of multidirectional (i.e. top-down and bottom-up) flows of influence, including broad jurisdictional variability in the way that systems for reporting mental health performance information are constructed [7]. Scholars have argued that this is illustrative of a form of governance termed decentralised integration [7].

The dynamics between these two tiers of government has two key implications for an emergent evidence base for Australian mental health policy: (1) it complicates the direction of causation (in terms of how Federal and State Governments influence each other and the health system) that may be inferred from evaluations; and (2) differences in routine data collection between jurisdictions affect what can be evaluated and how easily comparative data can be obtained.

Even within a single level of government (e.g. Federal Government), the concurrent application of multiple policy levers to achieve a desired outcome creates analytical difficulties for evaluation of causative factors. Policy levers are the tools or instruments governments use to achieve system-wide change. In the health sector, one accepted typology identifies five policy levers: organisation, regulation, community education, finance and payment [8, 9]. The combined use of multiple policy levers is commonplace and not incidental. For example, influencing the health system via organisational reform will likely have cost implications and thus necessitate the use of complementary finance and/or payment mechanisms. It may also potentially require the passage of new legislation (regulation) to invest the new organisation with legally recognised roles and responsibilities.

The feasibility of evaluation will vary at the individual program level, with some kinds of initiatives and outcomes lending themselves to policy experiments better than others. Nonetheless, the inability to empirically disentangle the effects of certain levers and/or activities, limits the applicability and validity of standard experimental designs [10, 11]. In general, evaluations based on experimental designs may provide only a partial answer to questions of policy success or failure.

### Political context

A range of bureaucratic, socio-political and ideological processes may also influence attributions of policy success and failure by framing the kinds of policies that are made and implemented, and how they are evaluated and interpreted. This context is not usually subject to evaluation [12, 13] and includes the broader influence of neoliberal theories and practices of governance, such as those embodied in New Public Management (NPM).

The advent of NPM in Australia saw a shift away from government-run monopolistic service provision, towards a more market-oriented approach [12, 14]. This paradigm shift and the resultant decades of reforms were based upon the belief that competitive tendering and private sector management would improve service efficiency, and improve the ability of the public sector to meet the needs of its constituents [12, 14, 15]*.*

Previous analysis of policy levers used in mental health [8] suggests that Australia’s mental health reforms are consistent with the NPM paradigm. This study found, for example, an increasing use of financing and payment levers over time, including the increased provision of funds to non-government service providers. The transition from a Federal Government-led National Community Awareness Program, towards one that was directed by not-for-profit service providers such as *beyondblue* and others [8], indicates a belief that the non-government organisation (NGO) sector can best meet the needs of mental health consumers. An observable shift toward formal incentive structures (payment) (e.g. the Better Outcomes in Mental Health Care and Better Access to Psychiatrists, Psychologists and General Practitioners through the Medicare Benefits Scheme initiatives), in lieu of non-tied funding (finance) [8], also conforms to the NPM paradigm and private sector managerial approach.

Devolving responsibility for services to the NGO sector has additional implications for the evaluation of centrally driven (Federal Government) reforms, such as the NMHS.

First, in a system where services are devolved to private providers, it becomes more complex to evaluate outcomes across the system. Further, critics argue that an unintended consequence of NPM has been the creation of an ‘audit society’. The preoccupation with target-setting and assessment with respect to key performance indicators (KPIs) is said to inadvertently compromise (rather than enhance) the quality of services offered to citizens [15, 16].

Finally, in some cases, the political context and timing of evaluations may prevent the clear and transparent evaluation of programs and policy – particularly with respect to failures [5, 15]. Howlett [6] has argued that, in a political culture that is driven by blame avoidance, understanding reasons for policy failure is a particularly important yet challenging area for study.

### A framework for assessing policy success and failure to aid in the selection of policy levers

Theory-based approaches to evaluation, and approaches that illuminate the contexts and mechanisms of change, provide an alternative to experimental approaches to policy learning [10, 17, 18]. Howlett [6] provides a framework for understanding policy success and failure that combines political and empirical elements in evaluation. As Table 1 indicates, Howlett (Howlett [6], drawing upon McConnell [1, 2]) proposes that attributions of ‘success’ and ‘failure’ go beyond outcome and process indicators and can refer to a range of different kinds of achievements or failures. Success could mean that a process was carried out as intended, irrespective of its broader or ultimate outcome (e.g. it could highlight that a successful review of human rights legislation was conducted). Alternatively, success could refer to the realisation of a positive outcome (e.g. that measurable reductions in socioeconomic inequality among minority groups occurred over a political term) (Howlett [6], drawing on McConnell [1, 2]).

**Table 1:** Multidimensional characteristics of policy success and failure (adapted from Howlett [6], drawing on McConnell [1, 2])

Evaluative Measure	Evidence of Success	Evidence of Failure
Original objectives	Objectives achieved	Objectives not achieved
Target group impact	Perceived positive impact	Perceived negative impact
Results (i.e. outcomes)	Problem improvement	Problem worsening
Significance	Important to act	Failing to act
Source of support/opposition	Key groups support	Key groups oppose
Jurisdictional comparisons	Leading or best practice	Someone else is doing better elsewhere
Balance sheet	Benefits outweigh costs	Costs outweigh benefits
Level of innovation	New changes	Old response
Normative stance	Right thing to do	Wrong thing to do

In the absence of measurable change, an initiative might be considered successful if key interest groups are satisfied that appropriate steps were taken to address their concerns (Howlett [6], drawing on McConnell [1, 2]). Success can also be considered in relative rather than absolute terms, by comparing what was done previously or what is done elsewhere. In this way, success may be assessed relative to the perceived efficacy of a range of alternative or proposed solutions that are widely known. Finally, policy successes can be linked to innovative measures such as a country’s inauguration of a mental health commission, or being the first country/ruling party to implement a particular model of service provision.

### Aims of this study

This paper investigates whether a theoretically-informed analysis of the successes and failures of past policy initiatives can assist in appraising the application of policy levers to mental health reform. This research aims to assist both policy makers and researchers in bridging the evidence-policy gap via the evaluation and targeted selection of appropriate policy levers.

## Methods

### Case study and documents

Our analysis centres on documentary analysis of policy lever usage under the Australian National Mental Health Strategy (NMHS). The NMHS, commencing with the publication of the first National Mental Health Plan in 1992, was a series of Federal Government publications that set the future direction for Australian mental health policy and service delivery reform. The four most influential of these publications are considered to be the first and second Plans, the Council of Australian Government (COAG) Plan and the 2011–12 Budget [8]. This is because the third and fourth National Mental Health Plans were reportedly overshadowed in terms of resource investment by the whole of government National Action Plan on Mental Health (the COAG Plan). Grace et al. [8] summarised national reform initiatives and key deliverables of the first and second National Mental Health Plans, COAG Plan and 2011–12 Budget, in accordance with the policy levers used in their implementation.

The present analysis focuses on the content and approach of the government-funded, independently conducted, evaluations, associated with the first three of these publications (evaluation documents listed below); noting there is no comparable formal government-funded evaluation for the 2011-12 Budget. Progress report IV was considered the primary evaluation document for the COAG Plan, with reference made to progress reports I-III, where they provide further elaboration on particular policy initiatives.

1. Evaluation of the National Mental Health Strategy [19]
2. Evaluation of the Second National Mental Health Plan [20]
3. National Action Plan for Mental Health progress reports I-IV [21–24]

### Analysis

FG first summarised the methodologies for each of the six evaluation documents in terms of their intended aims, actual focus and data sources used. The nine dimensions of success and failure, summarised in Table 1 *,* as proposed by Howlett (Howlett [6], drawing on McConnell [1, 2]), were then applied to the evaluation documents, to facilitate an analysis of policy levers used to implement the NMHS.

FG tabulated key initiatives and their evaluation for each Plan, grouped in terms of the lever(s) applied [8, 9] and the thematic priority area of mental healthcare that they addressed (*Human Rights and Community Attitudes, Responding to Community Need, Service Structures, Service Quality and Effectiveness, and Resources and Service Access)* [8, 25]. FG and CM then independently ascribed labels of success and failure to each initiative, according to the typology proposed by Howlett (Howlett [6], drawing on McConnell [1, 2]). These data were tabulated and synthesised to identify patterns in success and failure over time, and with respect to each of the five policy levers: regulation, community education, financing, payment and organisation.

Thirdly, FG and CM extracted a list of ‘unequivocal’ successes and failures, as depicted in the documents analysed. An unequivocal success was defined as the application of a policy lever to a particular initiative that met all measures of success by which it was evaluated and showed no measures of failure. Unequivocal successes were further categorised as being either ‘quick wins’ (i.e. those achieved within the life of a single Plan) or ‘cumulative successes’ (i.e. those spanning two or more Plans). An unequivocal failure was taken to be the application of a policy lever that met all measures of failure by which it was evaluated and achieved no measures of success.

## Results

### Evaluation scope and approach over time

The overall approach to evaluation varied across the NMHS. There was an observable shift in the approach to evaluation and a narrowing of scope over time. Table 2 presents an overview of these changes, outlining the intended aim, actual focus and data sources for each evaluation report.

**Table 2:** Intended (and actual) methodologies employed by Formal Government evaluations over the course of the NMHS

	First Plan	Second Plan	COAG Plan
Intended Aim	**O; I-**﻿Focus on reform approach rather than outcomes **﻿N-**﻿ Appropriateness of initiatives (normative stance)	**﻿O; I; R-﻿** Focus on reform approach as well as outcomes **N﻿﻿-﻿﻿** Appropriateness of initiatives (normative stance)	**O﻿-﻿** Focus on outcomes **R**﻿**-﻿** Performance against 12 population indicators
Actual Focus	**O﻿-**﻿Approach **R**﻿**-﻿** Problem improvement **TG﻿-﻿** Consumer perspectives	**O; I﻿-﻿** Approach **R﻿-﻿** Problem improvement **TG﻿-﻿** Consumer perspectives	**O**﻿**-﻿** Realisation of original objectives **R﻿-﻿** Problem improvement
Data Sources	• Repurposing of available national datasets • In-depth case studies • National stakeholder survey and consultations • International expert commentary	• Repurposing of available national datasets • National stakeholder survey and consultations • International expert commentary • Review of mental health by Mental Health Reference Group	• Repurposing of existing and new national datasets to report on 12 population indicators • Jurisdictional data (budget allocations and funding commitments)

Key: ​E﻿valuation Measure: *O* Objectives, *I* Innovation, *N* Normative Stance, *R* Results, *TG* Target Group Impact

Bold letters are used to indicate the evaluation measures

### First plan

The formal evaluation report for the first Plan explicitly stated that most of its objectives were focused on the process of reform, rather than what it achieved [19]. Compared with subsequent Plans, the first Plan’s objectives were quite ambitious and less clearly defined [8, 19].

In outlining their evaluation approach, the authors acknowledge that, at the time of the evaluation, specific outcome measures for each of the Plan’s objectives were not routinely collected. Instead, four research elements were chosen and used together to address the key outcome questions in the first Plan’s evaluation.

The first of these elements consisted of four in-depth community case studies, featuring consumer, carer, staff and external organisation perspectives on service changes made in area-based mental health services [19]. The document explicitly stated that the four communities were selected because they most closely “approximated the type of service models promoted by the NMHS, rather than being typical of the average services currently available” ([19], p7).

The other three research elements in the first evaluation were (1) national data sets (including source data for the inaugural National Mental Health Report), (2) findings from a national stakeholder survey, involving 182 national organisations representing health professionals, carers and consumers, and (3) international commentary, provided by a single expert, the Deputy Director of the Center for Mental Health Services, United States Department of Health and Human Services [19].

The first evaluation claimed to be process-oriented, but the evaluation predominantly assessed the degree of implementation (original objectives), problem improvement (results) and the target group’s perception of its perceived impact. Additionally, in describing the approach to evaluation, the authors claimed to provide an assessment of the appropriateness (normative stance) of initiatives in terms of consumer and carer needs. This was achieved in part through findings from a national stakeholder survey. However, there was little critical analysis of whether the initiatives were perceived to be the right or wrong thing to do.

### Second plan

Objectives under the second Plan were also quite ambitious. They included proposed improvements to service provision in rural and remote areas and for special needs populations, as well as improvements in overall service quality and standards [20].

Data collection methods were similar to those used for the first Plan. Relevant national data were presented alongside national community consultations and international expert commentary [20]. Instead of in-depth community case studies, the fourth research element included in the evaluation report was a review of mental health in the Australian Health Care Agreements, undertaken by an expert Mental Health Reference Group [20].

The second Plan’s evaluation report focussed on appraising implementation progress (original objectives) and problem improvement (results), while taking account of constraints on the sector and health system more broadly [20]. As with the first Plan, the authors proposed to evaluate each initiative according to its appropriateness (normative stance) for mental health consumers by presenting the results of stakeholder consultations alongside other evaluation data [20]. Again, however, specific discussion of the appropriateness of particular initiatives was not included in the evaluation report.

### COAG plan

The COAG Plan was the largest collective investment in mental health by Australian Governments, broadening the focus beyond health service reform to consider other sectors such as employment, education and correctional services, in recognition of their important contributions to the mental health of Australians.

Importantly, the COAG agenda was managed within the Department of Prime Minister and Cabinet instead of the Federal Health Minister’s portfolio, which had co-ordinated earlier Plans. Consequently, the evaluation approach differed markedly from those of the First and Second Plans. Twelve population-based indicators were developed to monitor progress under the COAG Plan. These indicators sought to evaluate problem improvement (results) in terms of health needs and service delivery changes, as well as broader changes to population health outcomes and social and economic wellbeing [24].

The evaluation approach used in COAG was the most restrictive of the three Plans. The authors’ focussed on allocations and funding commitments relating to the Plan’s original objectives, as well as results or progress as evidenced by the twelve progress indicators. No reference was made to alternative evaluation measures, such as target group impact, normative stance, or innovation [24]. Furthermore, unlike the evaluation reports presented for the first and second Plans, the COAG progress reports did not utilise stakeholder consultations, area case studies or international expert commentary.

Where an objective was reported to have been partially met, the COAG evaluation focused on reminding stakeholders of the existence of ongoing and/or proposed initiatives that would help to address the identified need. This differed from evaluations of the first and second Plans that sought to explain the lack of success and/or to offer policy learnings.

### Evaluation of policy levers over time

The evaluation of policy levers is presented for each of the three Plans (*refer* Tables 3, 4 and 5). Each table describes (1) the policy levers used; (2) reform priority area as categorised in our previous analysis [8]; and (3) attributions of success and failure identified in the documents. Where possible, COAG progress indicators relating to each of the original objectives are identified in italics in Table 5.

**Table 3:** Attributions of ‘Success’ and ‘Failure’ in relation to first National Mental Health Plan initiatives

First National Mental Health Plan				
Policy Lever	Policy Objective	Proposal(s)	Success	Failure
Organisation	*RCN* Involve consumers/carers in policy review and formulation	Formalise the inclusion of consumers and carers within working committees	**O-** Met to some degree **R-** Improvement in formalised participation	**O-** Met for only half of public sector organisations Not translated to private sector **R-** Participation but not leading to intended ‘good’ outcomes in terms of respect **TG-** Public/political dissatisfaction
	*SS* **Mainstream mental health service management**	Merge mental health into mainstream health management	**O-** Substantively met **R-** Mainstream management arrangements adopted across all jurisdictions	
	*SS* Shift acute beds to general hospitals	Shift psychiatric beds from stand-alone facilities to general hospitals	**O-** Substantively met **R-** Decrease in use of hospital-based services Funding shifted to community service sector	**R-** Resource reallocation and service availability is variable across jurisdictions Little adoption of population-based funding model to facilitate resource transfer **TG-** Community and public dissatisfaction High reports of areas of unmet need
	*SS* **Improve access to community crisis services**	Increase ambulatory workforce	**O-** Met **R-** Significant increase in ambulatory workforce	
	*SS* Improve coordination of care across service providers	Introduce case management system	**O-** Partially met – system introduced	**R-** Under-utilisation of case managers service Little measurable improvement in continuity of care **TG-** Community and public dissatisfaction
Regulation	*HR&CA* Reduce discrimination and stigmatisation of mental health consumers	Review anti-discrimination legislation	**O-** Substantively met **R-** Improvement in anti-discrimination legislation.	**TG-** Public/political dissatisfaction
	*HR&CA* **Adhere to UN Resolution 9B and Mental Health Statement of Rights and Responsibilities**	Review consumer rights and responsibilities as per State/Territory and Federal legislation	**O-** Substantively met (or in progress) **R-** Improvement in State/Territory and Federal legislation	
	*R&SA* **Simplification of cross-border treatment**	Identify and remove cross-border anomalies in diagnosis and treatment		**O-** Not met **R-** No change in cross-border anomalies **TG-** Low impact
	*SQ&E* Improve service quality and standards	Introduce nationally consistent standards for mental health care	**O-** Met **R-** Standards adopted across all jurisdictions Quality assurance programs introduced in some jurisdictions	**O-** Considerable ongoing development work required to see Standards fully accepted and implemented across all jurisdictions
	*SQ&E* Introduce independent evaluation body	Introduce an independent evaluation steering committee	**O-** Met **R-** Independent evaluation steering committee and National Mental Health Commission established	
	*SQ&E* Ongoing accountability and evaluation	Publish progress within annual Mental Health Reports Develop a National Mental Health Information strategy and minimum data set	**O-** Substantively met (at least for inpatient services) **R-** Accountability standards used as an example for other public policy	**O-** Not met for community based services (no minimum data set) **R-** No qualitative measure of ‘accountability’ No outcome measures yet recorded to evaluate intervention effect Routine assessment established in very few mental health centres
	*SS* Improve coordination of care across sectors	Review of interagency protocols	**O-** Substantively met	**R-** Under-utilisation of case managers service Little measurable improvement in continuity of care Not translated to local service level **TG-** Community and public dissatisfaction
Finance	*R&SA* Increase mental health budget	Increase recurrent mental health spending for Federal and State/Territory Governments	**O-** Substantively met **R-** Funding increases observed	**R-** Variable increase in funding across jurisdictions
	*R&SA* **Increase community-based and general hospital funding**	Increase community-based and general hospital funding for mental health	**O-** Substantively met **R-** Funds shifted to community service sector Significant increase in non-institutional spending	
	*R&SA* **Modify funding allocations for mental health**	Review Medicare Agreements	**O-** Substantively met **R-** Agreements more clearly outline bilateral funding arrangements	
	*SQ&E* Ensure fiscal accountability for mental health spending	Create a separate budget for mental health	**O-** Met	**R-** Funding continues to be allocated on historical basis Mental health sector-specific outcome-based funding tools remain underdeveloped and under-utilised
Community Education	*HR&CA* Improve mental health literacy (general public)	National Community Awareness Program	**O-** Partially met **R-** National community awareness program implemented	**O-** No substantial benefit achieved **R-** No measurable change in attitudes **TG-** Public dissatisfaction Approach not appropriate for minority groups No opportunity for local groups to coordinate promotional activity with the national campaign

Key: Reform Priority Area: *HR&CA* Human Rights and Community Attitudes, *RCN* Responding to Community Need, *SS* Service Structures, *SQ&E* Service Quality and Effectiveness and *R&SA* Resources and Service Access; Evaluation Measure: *O* Objectives, *R* Results, *I* Innovation, *TG* Target Group Impact; Unequivocal Successes and Failures appear in bold

Bold letters are used to indicate the evaluation measures

**Table 4:** Attributions of ‘Success’ and ‘Failure’ in relation to second National Mental Health Plan initiatives

Second National Mental Health Plan				
Policy Lever	Policy Objective	Proposal(s)	Success	Failure
Organisation	*RCN* Formalise consumer/carer consultation	Increase public and private sector organisations with formal consumer/carer consultation	**O-** Substantively met	**R-** Low level of genuine involvement or consultation **TG-** Public/consumer dissatisfaction (perceived change as insufficient)
	*R&SA* Increase early intervention services for youth	Provide specialist centres for youth early intervention, including assessment and treatment	**O-** Partially met **R-** Specialist centres and early intervention services increased	**R-** Not all groups’ needs met **TG-** Not all groups’ needs met
	*R&SA* **Improve service provision for special needs populations**	Develop new specialised service models		**O-** Not met **R-** Interventions underdeveloped Needs of CALD population not met Lack of service integration **TG-** Consumers not satisfied with level of change achieved/scale of impact
	*R&SA* Improve mental health curricula for Indigenous health workforce	Improve mental health curricula for Indigenous health workforce	**O-** Partially met **R-** Mental health curricula and culturally appropriate service models developed	**O-** Not met **R-** Shortage of health workers Ongoing need to improve links with mainstream services
	*SS* **Shift acute beds to general hospitals**	Relocate beds in stand-alone facilities to general hospitals	**O-** Substantively met **R-** Beds from stand-alone facilities relocated to general hospitals	
	*SS* Improve coordination of care across service providers	Formal protocols and agreements to support continuity of care	**O-** Partially met – system introduced	**R-** Under-utilisation of case managers service Little measurable improvement in continuity of care **TG-** Community and public dissatisfaction
Regulation	*SQ&E* Increased accountability for reform outcomes	Develop and apply new outcome measures	**O-** Partially met	**R-** Mental health workforce remain reluctant to participate in routine outcome monitoring **TG-** Consumers dissatisfied with progress
	*SQ&E* Improve service quality and standards	National Standards for Mental Health Services	**O-** Partially met	**R-** Mental health workforce remain reluctant to participate in routine outcome monitoring **TG-** Consumers dissatisfied with progress
	*SS* Improve coordination of care across sectors	Review interagency protocols to support continuity of care	**O-** Partially met	**R-** Lack of accountability Inadequate progress
Finance	*R&SA* Increase Federal and State/Territory expenditure on mental health	Review allocations under the general health budget for Federal and State/Territory Governments	**O-** Partially met	**R-** Size of sector did not increase Variable spending across jurisdictions
	*R&SA* Increase the size of community-based service sector	Grow 24-h staffed community based residential services Increase government spending on community services	**O-** Partially met	**R-** Low increase in services compared with closure of hospital services **TG-** Consumers report dissatisfaction with improvements
Community Education	*HR&CA* Reduce discrimination and stigmatisation of mental health consumers	Review media portrayal of mental illness	**O-** Partially met **R-** Review conducted Strategies implemented **I-** Use of innovative approach to engage general public in monitoring	**R-** No outcome measures collected to compare with baseline
	*HR&CA* Improve mental health literacy (health workers)	Provide mental health training to frontline workers	**O-** Partially met **R-** Education materials developed Strategies implemented	**R-** Consumers and carers continue to experience stigmatisation and discrimination (by professionals both inside and outside health sector)
	*HR&CA* Improve mental health literacy (general public)	Frontline service providers to distribute mental health brochures to patients Educate school children about mental health	**O-** Partially met **O-** Information material availability and distribution improved Community telephone survey suggested that people consider mental health to be a serious problem and to be more prevalent than in previous decades	**O-** Little or no substantial improvement in health literacy **R-** Materials do not suit all groups (i.e. exclude CALD and other minority groups)
Payment	*SS* **Ensure better links between primary and secondary providers**	Introduce new funding models Review existing MBS items	**O-** Substantively met	
	*R&SA* Improve service provision in rural/remote areas	Introduce new specialised funding models	**O-** Partially met **R-** Increase in dedicated service programs Improvement in recognition of special needs of this group	**O-** Most of NMHS objectives tailored to metropolitan areas **R-** Service gaps and workforce shortages in rural and regional areas remain

Key: Reform Priority Area: *HR&CA* Human Rights and Community Attitudes, *RCN* Responding to Community Need, *SS* Service Structures, *SQ&E* Service Quality and Effectiveness and *R&SA* Resources and Service Access; Evaluation Measure: *O* Objectives, *R* Results, *I* Innovation, *TG* Target Group Impact; Unequivocal Successes and Failures appear in bold

Bold letters are used to indicate the evaluation measures

**Table 5:** Attributions of ‘Success’ and ‘Failure’ in relation to COAG National Action Plan initiatives

COAG National Action Plan				
Policy Lever	Policy Objective	Proposal(s)	Success	Failure
Organisation	*R&SA* **Improve youth mental health services**	Review and consolidate existing youth mentoring and early intervention programs into a single program Initiate new youth early intervention projects	**O-** Substantively met	
	*R&SA* **Offer increased support for carers and families of people with mental illness**	Introduce a new Family Mental Health Support Service Increase mental health respite services for carers	**O-** Substantively met **R-** Increase in respite places Increase in education and formal support services	
	*R&SA* Greater employment and day-to-day living support for the mentally ill *Indicators 9 and 10*	Increase places in day-to-day living support programs and Personal Support program Increase number of Personal Helpers and Mentors Introduce Disability Employment Services group	**O-** Substantively met Disability Employment Services Group introduced **R-** Increase in funding and service provision	**R-** Employment rates remain low among the target group
	*R&SA* **Increase mental health workforce**	Increase the number of supported places in university mental health degrees, particularly to Indigenous students Specific funding targeted towards increasing Indigenous health workforce	**O-** Substantively met **R-** Increase in supported places and scholarships for formal training	
	SS **Improve and integrate drug and alcohol services within broader mental health services**	Provide additional funding to drug and alcohol service providers	**O-** Substantively met **R-** Funding increased, grants awarded to NGOs	
	*R&SA* **Increase service coverage in rural/remote areas** *Indicator 5*	Introduce a 24-h 7 day mental health telephone service Increase web-based mental health resources	**O-** Substantively met Flexible service delivery modes introduced (telephone, online services)	
	*SS* Improve coordination of care *Indicator 7*	Introduce step-up and step-down community facilities Utilise community coordinators	**O-** Partially met **R-** Principles and implementation guidelines developed	**R-** Variable progress in jurisdictions Lack of consistent approach or outcome Lack of accountability
Regulation	*SQ&E* **Increase consultation between State/Territory and Federal Governments**	Establish COAG Mental Health Groups in each jurisdiction	**O-** Substantively met	
	*SQ&E* **Increased accountability for reform outcomes**	Publish official progress reports annually	**O-** Substantively met	
Payment	*R&SA* **Improve service provision in rural/remote areas**	Use of flexible funding models to improve access to allied and nursing mental health services in rural and regional areas	**O-** Substantively met	
	*R&SA* **Increase health workforce in rural and remote areas, particularly mental health nurses** *Indicator 5*	Introduce Mental Health Nurse Incentive Program Introduce flexible employment schemes for rural and regional areas	**O-** Substantively met Incentives and flexible employment schemes introduced	
	*SS* **Improve links between primary and secondary providers** *Indicator 5*	Introduction of new MBS items to support referral between health practitioners	**O-** Substantively met	
Community Education	R&SA **Review mental health content in tertiary health degrees**	Review mental health content in tertiary health degrees	**O-** Substantively met **R-** Final project reports identify an increased focus on mental health in both theoretical and clinical subjects	

Key: Reform Priority Area: Human Rights and Community Attitudes (HR&CA), *RCN* Responding to Community Need, *SS* Service Structures, *SQ&E* Service Quality and Effectiveness and *R&SA* Resources and Service Access; Evaluation Measure: *O* Objectives, *R* Results, *I* Innovation, *TG* Target Group Impact; Unequivocal Successes and Failures appear in bold

Bold letters are used to indicate the evaluation measures

Three types of success were identified, relating to objective, results, and innovation. Similarly, three kinds of failure were identified, relating to objective, results and target group impact. The remaining five dimensions of success and failure, as described by Howlett (Howlett [6], drawing on McConnell [1, 2]), were not identified in any of the formal evaluation reports studied.

The evaluation measures applied to particular policy levers varied over the course of the NMHS. This variability in evaluation approach is described in the following sections, with reference to the policy lever being evaluated.

### Organisation

Under the first and second Plans, organisational levers elicited the most nuanced, multidimensional analyses, with both successes and failures evaluated against their original objectives, results and target group impact. The evaluation reports provided a more detailed appraisal of successes and failures of organisational initiatives. Where appropriate, they offered explanations for *why* particular programs were unsuccessful and how future reform should be modified to better address the original objectives.

This kind of detailed analysis was provided in the appraisal of the policy initiative to “involve consumers/carers in policy review and formulation” from the first Plan (*refer* Table 3). The evaluation found that public sector progress had surpassed that of the private sector [19]. The authors suggested that in focusing its approach on the incentives and accountabilities relevant to Federal, State and Territory Governments, the NMHS may have failed to affect change outside of the public sector ([19], p14).

The objectives of the COAG Plan were more clearly defined than those of the first and second Plans, and their achievement tended to be evaluated using a single discrete (mostly quantitative) outcome indicator. This was particularly notable for organisation proposals; for example, the provision of additional support to carers and families of people with mental illness was evidenced by an increased number of available respite places and the introduction of the Family Mental Health Support Service (*refer* Table 5) [24].

### Community education

Direct government involvement in community education decreased over the course of the NMHS with the adoption of an NGO-driven model [8]. There was one initiative that utilised community education in the first Plan, three in the second Plan and one in the COAG Plan.

The community education lever was primarily evaluated against objectives and results. There was one instance, under the second Plan, of success being evaluated by the degree of innovation in the reform approach. This appraisal commented on the Federal Government’s “world-leading” approach to reducing discrimination and stigmatisation of mental health consumers, achieved in partnership with the media ([20], p2, p16).

### Regulation

The use of regulation as a policy lever was most prominent in the first Plan, in which seven objectives relating to regulatory changes, compared to three under the second Plan and two under the COAG Plan. The first Plan featured a number of clearly defined regulatory objectives such as a review of anti-discrimination legislation and consumer rights and responsibilities, and the introduction of an independent evaluation body (*refer* Table 3 *)* [19]. In the second and COAG Plans, the proposed regulatory changes attempted to achieve more ambitious high-level system improvements, such as improving service quality and government accountability for reform outcomes (*refer* Tables 4 and 5 *).*

Both success and failure were evaluated against original objectives, with failure also evaluated in terms of target group impact. Notably, there was no attribution of failure with respect to regulatory objectives under the COAG Plan.

### Finance and payment

Finance and payment objectives were applied in a more discrete and quantifiable manner than the organisational and community education levers. There was an increase in the use of monetary levers, and payment in particular, over the course of the NMHS [8]. Examples included new funding models and incentives that were introduced in the second Plan and continued under the COAG Plan [20, 24]. These aimed to increase the availability of mental health services in rural and remote areas (*refer* Tables 4 and 5).

The evaluation of this lever was asymmetrical. Whereas success was evaluated in terms of the original objectives, failure was sometimes, but not always, evaluated in terms of results and target group impact.

### Case studies: unequivocal success and failure

The following sections describe objectives deemed to be unequivocal successes or failures. Unequivocal successes were further divided into those achieved within the life of a single Plan (quick wins) and those that were achieved over the span of two or more Plans (cumulative successes). Policy objectives considered as either unequivocal successes or failures appear in bold in Tables 3, 4 and 5.

### Unequivocal successes

#### Quick wins

There were six quick wins observable under the first Plan (*refer* Table 3). The first of these was the mainstreaming of mental health management into general area-based health services, using organisational and finance levers [19]. The organisational lever was also used to increase the ambulatory workforce and access to crisis care services [19].

A quick win for regulation was the commissioning of a systematic review of Federal and State/Territory legislation on consumer rights and responsibilities [19]. The Mental Health Statement of Rights and Responsibilities was amended to reflect UN Resolution 9B, to improve the respect accorded to mental health consumers [19]. This change was judged to be successful, when evaluated against the original objective, because reviews and legislative amendments had been made, or were in progress, across all jurisdictions [19]. Another successful application of the regulation lever was the establishment of an independent evaluation steering committee and National Mental Health Commission, responsible for the delivery of the first Plan’s formal evaluation report [19].

The finance lever was successfully used to increase community and mainstream hospital (i.e. non-institutional) funding. Funds released after deinstitutionalisation were reallocated toward community-based services at a Federal and State/Territory level [19]. Non-institutional spending grew by 55% (A$216 million). This funding was primarily used to increase ambulatory (crisis care) services (A$135 million) and acute services in mainstream/general hospitals (A$41 million).

The creation of a dedicated, separate national mental health budget and increases in overall portfolio expenditure (States/Territories by 6.3% and Federal by 61%), were all quickly achieved under the first Plan [19].

Under the second Plan, the only quick win was the introduction of new incentive structures (e.g. the Better Outcomes in Mental Health Care initiative and new Medicare Benefits Scheme (MBS) items). These facilitated referrals and case conferencing between primary care providers (GPs) and specialist mental health services [20].

The COAG Plan’s formal progress report identified four quick wins. The organisation lever was used to introduce a Family Mental Health Support Service, providing increased support and respite for carers and families of people with mental illness [24]. Drug and alcohol services were successfully integrated within broader mental health services by way of a A$74 million increase in funding, including new NGO grants.

The payment lever was successfully used to introduce new flexible employment incentives to attract mental health workers to work in rural and remote areas (e.g. Mental Health Nurse Incentive Program) [24]. Finally, the regulation lever was successfully used to introduce COAG Mental Health Groups in each jurisdiction, to improve consultation between Federal and State/Territory Governments [24].

#### Cumulative successes

Eight cumulative successes were observed across the course of the NMHS. The majority of these (seven) were achieved under the COAG Plan.

Under the second Plan, there was one cumulative success, namely the relocation of acute mental health beds from specialist psychiatric institutions to general hospitals. Following completion of the first Plan, 39% of acute beds were located in mainstream services and a further 41% were relocated under the second Plan [20].

Cumulative successes under the COAG Plan included the use of organisational levers to review and consolidate existing youth early intervention, mentoring and employment programs into a single case-management program, Youth Connections [24].

The second Plan saw the introduction of education and training programs to build capacity in existing health workers. Building upon these initiatives, the COAG Plan saw the introduction of educational places, scholarships and clinical training programs to further increase the capacity and reach of the mental health workforce [24].

MBS items for tele-psychiatry were originally introduced under the second Plan [20]. These were expanded to include specialised web- and telephone-based service models to deliver cognitive behavioural therapy under the Access to Allied Psychological Services program (ATAPS). This application of the payment lever was judged to be an unequivocal success (in terms of the realisation of original objectives) in the COAG Plan [24]. So too was the implementation of flexible funding models that aimed to increase access to allied and nursing mental health services, commissioned under the second Plan [20, 24].

Regulation was used to deliver the fifth cumulative success of the COAG Plan, through the continued publication of annual progress reports, albeit in a new format [24].

Building upon previous educational materials and strategies developed under the second Plan [20], the COAG Plan used the community education lever to review mental health content in tertiary health degrees for nursing, medical and allied health programs [24]. This initiative, which sought to improve the mental health literacy of health workers [24], was judged to be an unequivocal success.

The final cumulative success was the introduction of new MBS items and fee-for-service (payment) models to foster linkages between primary and secondary care providers. Commencing under the second Plan [20], this initiative was reformulated under the COAG Plan (e.g. the Better Access to Psychiatrists, Psychologists, and General Practitioners through the MBS (Better Access) initiative) where it was deemed successful [24].

### Unequivocal failures

There was one unequivocal failure reported under the first Plan, namely the use of regulatory mechanisms to simplify cross-border treatment. The evaluation reported that there was no change in jurisdictional anomalies in treatment [19]. This objective was subsequently omitted from the second and COAG Plans, although improved communication across Federal and States/Territories was facilitated through the organisational lever, through the introduction of COAG Mental Health Groups [24].

Under the second Plan, proposed changes to service provision for consumers with special needs was the only unequivocal failure [20]. Consumers were not satisfied with the change, reporting that interventions were underdeveloped and lacking in service integration. This resulted in failure to meet the needs of culturally and linguistically diverse populations [20]. As was the case with the other unequivocal failure, initiatives relating to developing better service models for consumers with special needs were absent from the subsequent (COAG) Plan [24].

No unequivocal failures were reported under the COAG Plan.

## Discussion

Our analysis highlights the variability in the character of evaluations both over time and in relation to different policy levers. This variability complicates the assessment of policy success and failure over the course of Australia’s NMHS.

Our analysis indicates that the last Plan (COAG) was the most successful of the three Plans analysed. One interpretation of this finding is that there was an overall improvement in the development and application of policy levers over time. However, there are other plausible explanations. It could be that this apparent success is the product of a reduction in the depth of evaluation overtime and/or differences in the frames of reference. Another possibility is that there was an increased imperative for evaluators to demonstrate progress, resulting in a ‘success bias’.

### Changes in depth of evaluation over time and increased success bias

The apparent increases in success could be indicative of policy learning in relation to setting and appraising objectives to maximise the chance of success, either practically or rhetorically. Our analysis found that both the formal government Plans and their evaluations became more refined over the course of the NMHS. Plans adopted more tangible and focussed objectives, for which achievement could be more easily demonstrated (or ‘ticked off’), and intractable issues were dropped from subsequent Plans.

The omission of stakeholder views from the COAG evaluation constitutes an important gap in understanding and a significant point of difference when compared with evaluations of previous Plans. Changes in the structure of the health system (organisation) and use of community education are perhaps the most publicly visible kinds of reform. Thus, they are initiatives that can be evaluated more easily by consumers in a way that other levers (e.g. regulation*,* finance etc.) may not.

Under the COAG Plan there was a notable increase in the use of finance and payment levers. These monetary levers are more directly linked with certain kinds of routine data collection and thus more easily appraised than other types of levers. The quantitative measurability of these policy levers, coupled with a decreased reliance upon qualitative outcome measures (e.g. consumer perspectives), may explain the tendency toward evaluations based on original objectives and results, rather than target group impact or innovation.

To address concerns about the possible negative consequences of a narrow KPI-based evaluative focus, it has been suggested that evaluations of services should include the concept of ‘public value’ to capture higher order aspirations e.g. trust, fairness, equity, legitimacy and confidence [14].

### Changing frame of reference for evaluation

The different frame of reference and evaluation approach used for the COAG Plan, compared with the previous two Plans, may also offer some explanation for the COAG Plan’s apparent success.

COAG represented an unprecedented commitment by both Federal and State/Territory Governments. It overshadowed the third Plan to give greater impetus and renewed government support for mental health reform [8, 24]. This form of collaboration across levels of government and service sectors is highly desirable in raising the profile of a reform agenda, increasing resource allocation and mitigating obstructive interdepartmental rivalries.

Our analysis suggests that a broadening focus, and the allocation of health reform to the Prime Minister’s portfolio, can also result in changes in the style and approach of formal government evaluations. In this case, it was found to result in a dilution and reduction in the depth of evaluations concerning the achievement of service delivery reform objectives.

Making inferences based on correlations between the twelve broad population indicators and the COAG Plan’s stated objectives is problematic. Aside from the standard difficulties of inferring causation from correlation, attempting to match indicators to specific reform objectives is hampered by the lack of direct correspondence between individual initiatives and population level outcomes. In this way, the COAG Plan’s evaluation approach precludes genuine policy learning.

### Policy learnings on success and failure

Despite such limitations, policy learnings can be derived from the unequivocal successes and unequivocal failures for each Plan. We identified eleven quick wins over the course of the NMHS. These objectives did not appear to be associated with any one policy lever or reform priority. There was, unsurprisingly, an inverse association between the degree of complexity involved in implementing a particular objective and its likelihood of success.

Proposals involving a single level of government, and those involving relatively straightforward changes to legislation, budget allocations or the organisation of existing services, were achieved in the course of a single Plan. Conversely, proposals requiring cooperation across jurisdictions or service sectors were less likely to meet their objectives or have the intended target group impact, and were thus reported as unequivocal failures.

In some cases, more ambitious objectives did appear to be achieved cumulatively over the course of the NMHS. This highlights the need to allow sufficient time to achieve policy success and also the fact that more challenging and complex health service reforms may take more than one Plan to achieve. Cumulative successes were commonly associated with a change in approach and/or the application of a different policy lever (e.g. payment), suggesting that complex health reforms may require some degree of trial and error and/or the coordination of different initiatives over time.

### Recommendations for future evaluations

The present documentary analysis centred on the application of a framework, adapted from Howlett (Howlett [6], drawing on McConnell [1, 2]), to the appraisal of policy lever success and failure. Whilst in the present study, this framework has been applied in the context of Australian mental health reform, it is likely to have broader applications across other areas of health and public policy.

The results of this analysis suggest that the context and frame of reference within which reform agendas are set, can have a significant effect on the choice of evaluation measures and the depth of analysis. We suggest that national level evaluations should aim for maximum consistency in the evaluation approaches that are used across successive government strategies. This would enable more meaningful inferences to be made about the successes and failures of each reform agenda and the effectiveness of particular policy levers, applied independently or in combination.

To maximise policy learning and improvement, there is a need to strike a balance between visionary and pragmatic approaches to agenda setting and policy evaluation. Our analysis suggests that policy objectives that are too ambitious can result in vagueness regarding their implementation, and in how initiatives are related to outcomes. The result is that such initiatives are harder to prove successful. Conversely, being myopic about what kinds of outcomes can be reasonably achieved over a short space of time and using overly technocratic evaluation approaches may appear to achieve greater success, but can also limit the scope of reforms, stifle innovation, and preclude genuine policy learning and improvement.

Our analysis indicated that attributions of success and failure were dominated by two kinds of measure: whether the original objectives had been met and whether these objectives had produced the intended set of outcomes (results). Whilst these are important aspects of evaluation, consideration should be given to the full range of evaluative measures (*refer* Table 1 *)* to better reflect the political, social, and jurisdictional contexts in which policy levers are applied, and to promote future innovation.

## Limitations and future research directions

The present analysis developed and employed a framework, based on theories from the field of policy studies Howlett [6], drawing on McConnell [1, 2]) to attribute success and failure to each of the NMHS’s objectives and proposals. This approach requires some subjective judgement, and despite attributions being confirmed by two independent authors, it is possible that others who apply these criteria may reach different conclusions.

Policy objectives and associated evaluation data were classified into discrete policy levers or instruments used to achieve system-wide change [8]. This approach does not permit formal qualification of the extent to which the success or failure of a given lever was dependent upon the simultaneous application of complementary or antagonistic policy levers.

Further insights could be gained by analysing a wider range of Federal and/or State and Territory program-specific evaluations, academic papers and media publications. Where available, these resources could help to identify contextual factors relevant to understanding why the application of particular policy levers succeeded or failed.

## Conclusion

This analysis represents an important first step in developing an evidence-base for mental health policy. It highlights the complexities of health service reform and underscores the limitations of narrowly focused empirical approaches. The theoretical framework presented in this analysis could be used to inform future health service evaluations, and may assist in the targeted selection of appropriate policy levers.

## Abbreviations

AHMAC: Australian Health Ministers Advisory Council
ATAPS: Access to Allied Psychologists Scheme
CALD: culturally and linguistically diverse
COAG: Council of Australian Governments
GP: General Practitioners
KPI: key performance indicators
MBS: Medicare Benefits Scheme
NPM: New Public Management
NGO: non-government organisation
NMHS: National Mental Health Strategy
